# Supplementary material for: Development of a computer-aided design software for the quantitative evaluation of aesthetic damage
Source: PLoS One. 2019 Dec 18;14(12):e0226322. doi: 10.1371/journal.pone.0226322 (PMC6919621; doi:10.1371/journal.pone.0226322)
Supplement: S1 Code site — 1. (ZIP) [file pone.0226322.s001.zip › [EESC_jr][Projeto]Relatorio_07_21/reports/templates_zip/gentelella-master/vendors/Flot/examples/axes-time/index.html]

Flot Examples: Time Axes


## Time Axes

Monthly mean atmospheric CO_2_ in PPM at Mauna Loa, Hawaii (source: NOAA/ESRL).

If you tell Flot that an axis represents time, the data will be interpreted as timestamps and the ticks adjusted and formatted accordingly.

Zoom to: Whole period
1990-2000
1996-2000

Zoom to: 1999 by quarter
1999 by month
Last week of 1999
Dec. 31, 1999

The timestamps must be specified as Javascript timestamps, as milliseconds since January 1, 1970 00:00. This is like Unix timestamps, but in milliseconds instead of seconds (remember to multiply with 1000!).

As an extra caveat, the timestamps are interpreted according to UTC and, by default, displayed as such. You can set the axis "timezone" option to "browser" to display the timestamps in the user's timezone, or, if you use timezoneJS, you can specify a time zone.

Copyright © 2007 - 2014 IOLA and Ole Laursen
